# Supplementary figures and images for: Detection of breast cancer lymph node metastases in frozen sections with a point-of-care low-cost microscope scanner
Source: PLoS One. 2019 Mar 19;14(3):e0208366. doi: 10.1371/journal.pone.0208366 (PMC6424449; doi:10.1371/journal.pone.0208366)

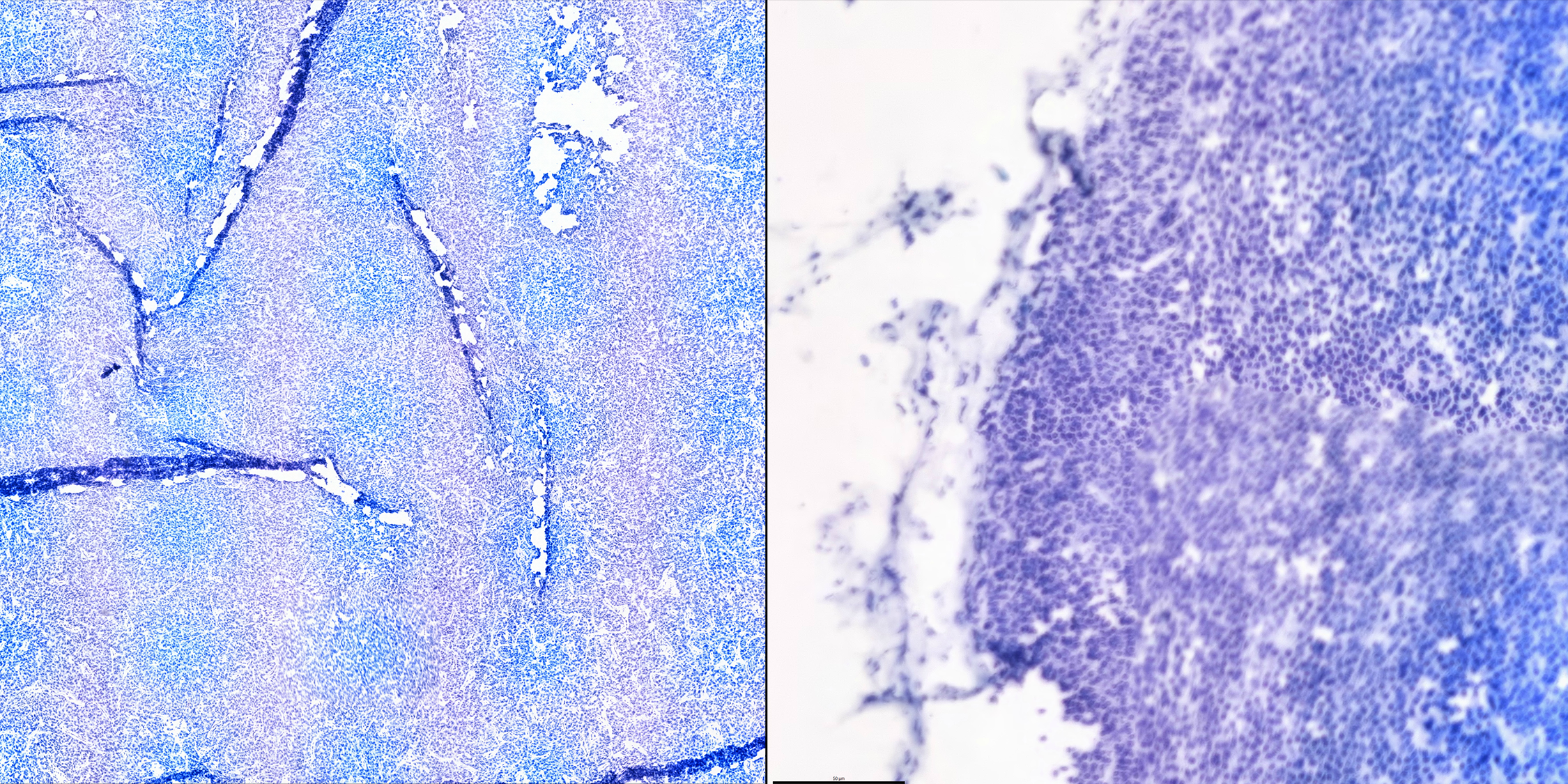

Supplement: S1 Fig — A small number of slides scanned with the miniature microscope scanner displayed variations in color and brightness, producing a grid of vignetting artifacts in the WSIs. This artifact was caused by incorrect compensation for lens color shading, i.e. correcting for light fall-off near edges of the field-of-view of the lens type used in the system. Normally, this is corrected for by software adjustments, using a blankfield image to calibrate for differences in brightness and color. Incorrect adjustment for lens color shading in images constituting the WSIs results in a characteristic, repeating pattern of “vignetting” in the final digital image (left panel). This problem was present in a small number of WSIs scanned with the miniature microscope scanner and could be solved by rescanning affected samples after calibrating software and updating blankfield images. Areas in some WSIs displayed focusing problems (right panel) due to the auto focus system focusing on the wrong plane. This occasionally affected small areas in some samples, scanned with both devices. Both problems mentioned here could corrected by rescanning affected slides or areas, after performing software adjustments and thus did not represent persistent issues. (TIF) [file pone.0208366.s001.tif]

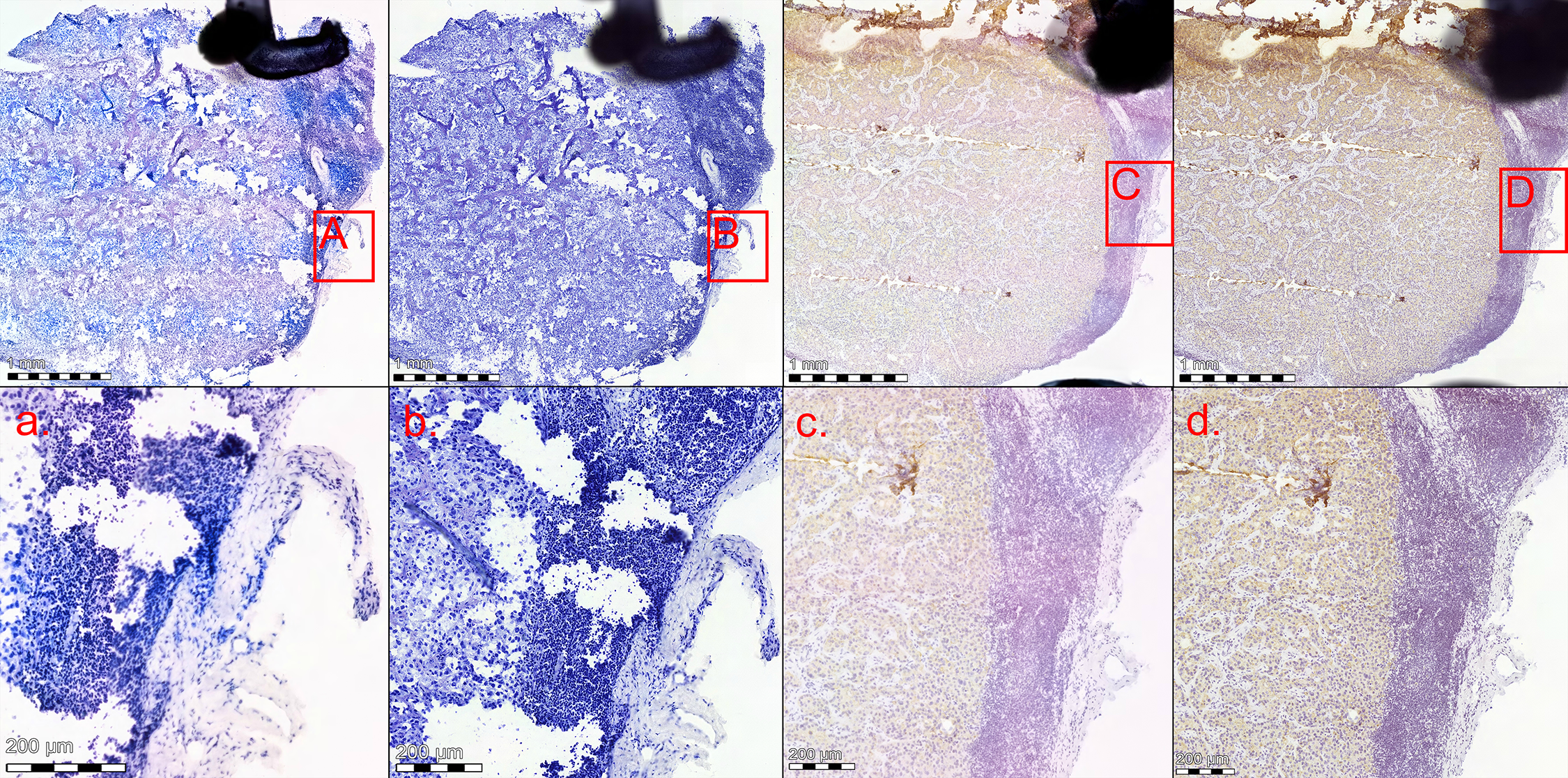

Supplement: S2 Fig — Lymph node frozen section with macrometastasis, stained with toluidine blue (left) and anti-cytokeratin (right) staining, and scanned with both devices. Upper images showing overview of the FS section and lower side showing enlarged areas (as indicated with red bounding boxes). Slides scanned with the miniature microscope scanner on left side (A. and C.), and reference slide scanner WSIs on the right side for comparison (B. and D.). (TIF) [file pone.0208366.s002.tif]
