# Supplementary material for: Detection of breast cancer lymph node metastases in frozen sections with a point-of-care low-cost microscope scanner
Source: PLoS One. 2019 Mar 19;14(3):e0208366. doi: 10.1371/journal.pone.0208366 (PMC6424449; doi:10.1371/journal.pone.0208366)
Supplement: S1 Document — (PDF) [file pone.0208366.s003.pdf]

Detection of breast cancer lymph node metastases in frozen sections with a point-of-care low-cost microscope scanner.

O. Holmstrom et al.

Manuscript for PLOS One. Supplementary information S3.

S3 Document. Questions and summary of feedback from pathologists on perceived image quality of the evaluated systems.

1. **Did you experience that scoring with the low-cost device was significantly affected due to lower image quality?**

    Image quality was not considered a significant factor in the sample analysis. Both pathologists considered the spatial resolution and imaging performance to be sufficient for reliable sample analysis and detection of metastases. Image quality was perceived as slightly lower, resulting in a blurrier image when viewing samples at high magnification with the low-cost scanner compared to the high-end scanner. Although this was not considered a significantly limiting factor for detection of larger lesions, it was speculated that higher imaging performance would be preferable to detect smaller lesions, such as ITC's and micrometastases (<2 mm) due to requirements of resolving more details at a sub-cellular level. It was also suggested that less time is spent analyzing this type of findings, as the clinical significance for these finding typically do not affect treatment of the patient, and thus these are more likely to not be detected by the pathologist. Although the general consensus was that spatial resolution was not a significantly limiting factor in this study, it was discussed that toluidine blue-stained sections might be easier to analyze with a device with higher spatial image resolution. Both

pathologists agreed that the analysis of ultra-rapid cytokeratin-stained sections was not a problem with samples scanned with the low-cost scanner.

2. **A lower spatial resolution is the major difference between the systems, i.e. ability to resolve fine detail in the samples at high magnification. Did you experience that the level of imaging performance of the low-cost scanner was still sufficient for reliable sample analysis in this study?**
   - The spatial resolution was considered sufficient for sample analysis and detection of metastases in this study, as discussed above. The lower resolution was noted when viewing samples at high magnification, but both experts agreed that the imaging performance was sufficient for the analysis of samples and detection of especially macro-metastases. The images from the miniature microscope scanner were described as “not as sharp” when zoomed in at high magnification, and occasionally focusing problems in parts of the images were present. Furthermore, variations in brightness and color (e.g. the repearing square artifacts of individual tiles) was noted in some images. A small number of samples displayed these problems, and they were not considered likely to have affected sample analysis significantly.

3. **The amount of false negative samples was slightly higher with the low-cost microscope. In your experience, what would be the most likely explanation for this?**
   - While the differences in image quality was considered possible to affect sample analysis, both pathologists agreed that this was unlikely to be a major cause for discrepancy. Instead it was suggested that the human error due to time constraints and possibly artifacts in the physical sample could have affected the analysis. The same explanations were considered as most likely

causes for the false positive samples with the low-cost device. Upon viewing the samples again, the correct diagnosis was made by the pathologist.

4. **In general, do you have other comments or thoughts about the images from the low-cost scanner compared to the high-end system?**
    - No further comments were raised here, the same factors as mentioned above were discussed.
